# Supplementary material for: TRPS1 modulates chromatin accessibility to regulate estrogen receptor (ER) binding and ER target gene expression in luminal breast cancer cells
Source: bioRxiv. 2023 Jul 3:2023.07.03.547524. Preprint. [Version 1] doi: 10.1101/2023.07.03.547524 (PMC10349936; doi:10.1101/2023.07.03.547524)
Supplement: 1 [file NIHPP2023.07.03.547524V1-supplement-1.pdf]

bioRxiv preprint doi: https://doi.org/10.1101/2023.07.03.547524; this version posted July 3, 2023. The copyright holder for this preprint (which was not certified by peer review) is the author/funder, who has granted bioRxiv a license to display the preprint in perpetuity. It is made available under aCC-BY 4.0 International license.

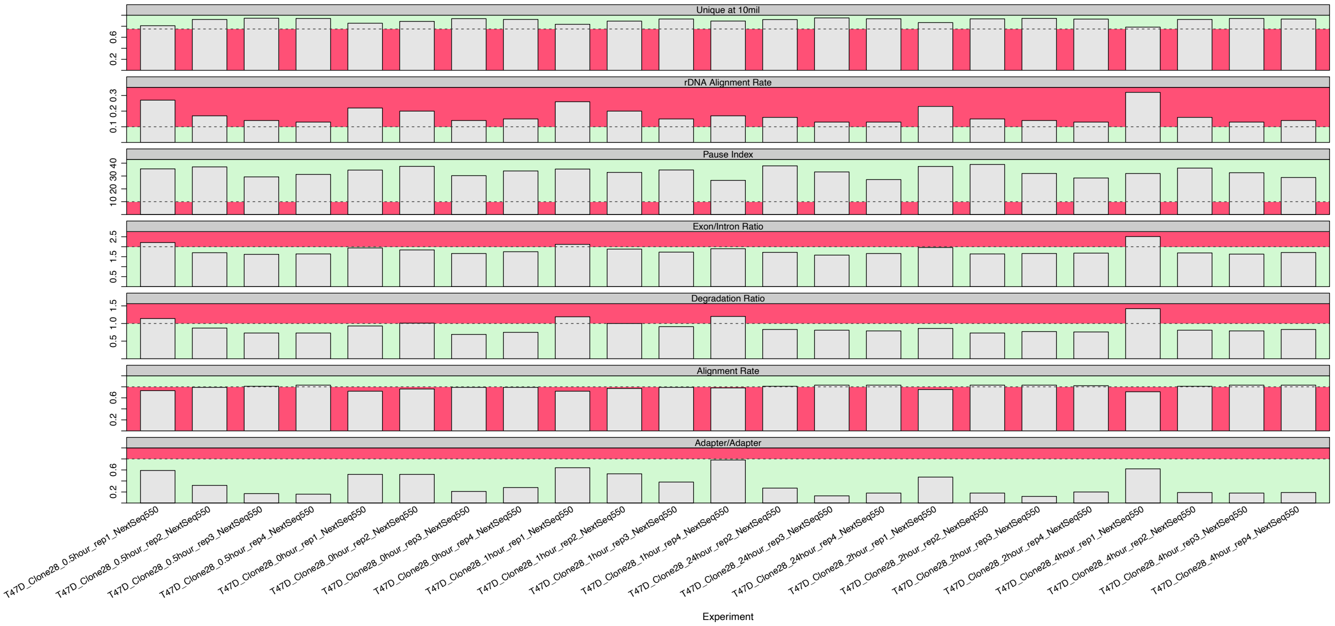

Fig. S1. Quality control metrics for PRO-seq libraries. Quality control metrics are defined as in (Scott et al. 2022). Each metric is a row, and each sample is a column. The green region for each metric is the goal for a high quality library.

bioRxiv preprint doi: https://doi.org/10.1101/2023.07.03.547524; this version posted July 3, 2023. The copyright holder for this preprint (which was not certified by peer review) is the author/funder, who has granted bioRxiv a license to display the preprint in perpetuity. It is made available under aCC-BY 4.0 International license.

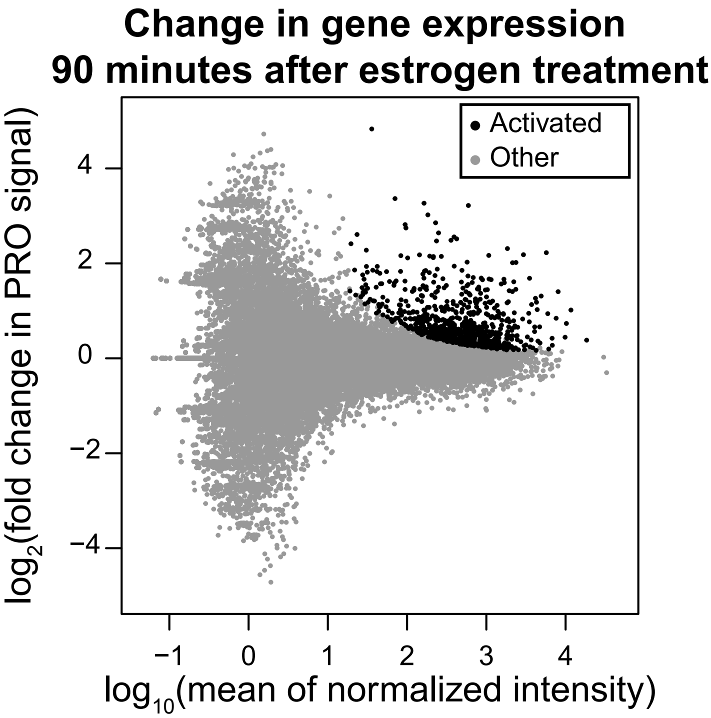

Fig. S2. Acute estrogen treatment identifies direct ER target genes in T47D cells. MA plot of PRO signal, with fold change values representing transcription in the 90 minute estrogen treatment condition relative to the DMSO condition. Each point represents a gene, and black points represent the estrogen-activated genes that we use in Figure 5.

bioRxiv preprint doi: https://doi.org/10.1101/2023.07.03.547524; this version posted July 3, 2023. The copyright holder for this preprint (which was not certified by peer review) is the author/funder, who has granted bioRxiv a license to display the preprint in perpetuity. It is made available under aCC-BY 4.0 International license.

Motif	Consensus	Percent increased	Percent unchanged	Padj
GATA5-Jaspar	WGATAASR	69.70	8.47	2.95e-88
GATA3-Jaspar	WGATAASR	62.29	6.99	6.34e-76
GATA3-Homer	AGATAASR	66.10	9.96	8.55e-74
TRPS1-Homer	AGATAAVANN	61.86	8.05	6.96e-71
GATA2-Jaspar	NBCTTATCTNH	54.45	5.30	1.76e-65
Gata6-Homer	BCTTATCWNN	56.78	6.99	9.67e-64
Gata4-Homer	NBWGATAAGV	54.24	6.57	4.83e-60
GATA6-Jaspar	NDNAGATAAGADD	48.52	5.08	2.20e-54
Gata2-Homer	BBCTTATCTS	46.19	4.24	2.54e-53
Gata6-Secondary-Uniprobe	WHNVDWGATAAGADTHN	45.13	3.81	4.37e-53
Gata1-Jaspar	TYCTTATCTSY	41.95	3.18	7.08e-50
Gata1-Homer	SAGATAAGVV	38.56	3.18	9.24e-44
Gata5-Secondary-Uniprobe	HWWRCTGATAAGRRNAN	40.47	4.66	2.71e-41
Gata4-Jaspar	YCTTATCTSHB	40.47	5.08	9.06e-40
Gata3-Secondary-Uniprobe	BDWDDAKAGATAAGARWTDARD	31.99	2.75	2.39e-34
TEAD3-Jaspar	RCATTCCW	39.83	11.02	7.01e-23
Mtf1-Secondary-Uniprobe	ANATAWGAAAWADV	49.79	18.86	1.19e-21
FOXP3-Jaspar	RTAAACA	48.94	18.22	1.31e-21
Arid3a-Secondary-Uniprobe	NNVVRTATCRDRWHD	29.87	5.93	5.62e-21
FOXK1-Homer	NNNTGTTTAY	47.46	17.58	9.71e-21
TEAD4-Jaspar	NRCATTCCWN	41.53	13.77	9.84e-20
HLTF-Jaspar	NHMCWTDKNN	52.54	22.46	1.75e-19
TEAD1-Jaspar	YRCATTCCWH	40.04	13.35	1.34e-18
FOXD2-Jaspar	GTAAACA	49.15	20.55	3.63e-18
FOXB1-Jaspar	WATGTAAATAT	31.78	9.11	2.21e-16
TEAD4-Homer	SSWGGAATGY	47.67	20.76	4.13e-16
Glis2-Secondary-Uniprobe	HDTATTAWTAAAGV	23.73	4.87	1.57e-15
FOXM1-Homer	TRTTTRCYYW	47.03	20.97	4.30e-15
Arid3a-Jaspar	ATYAAA	30.08	8.90	8.85e-15
FOXL1-Jaspar	RTAAACA	42.16	17.37	9.78e-15
Prop1-Homer	NTAATBNVATTA	27.97	7.63	9.94e-15
FOXC1-Jaspar	WAWGTAAAYAW	34.53	12.08	2.36e-14
TEAD2-Jaspar	NYACATTCCWNNS	38.56	15.25	7.03e-14
Sox5-Secondary-Uniprobe	NHDCATMATTDASNN	27.33	7.84	1.43e-13
FoxD3-Homer	TGTTTAYTTWGC	36.65	14.19	2.42e-13
Foxj2-Jaspar	RTAAACAA	31.57	10.81	3.93e-13
TEAD3-Homer	YRCATTCCAG	27.54	8.26	5.35e-13
Hoxa11-Homer	TTTTATDRCH	29.03	9.32	8.93e-13
NR2F2-Jaspar	NAAAGGTCANR	24.79	6.78	1.40e-12
FOXC2-Jaspar	WAWGTAAACAWW	36.65	14.83	1.88e-12
Zfp128-Secondary-Uniprobe	NGTATAYDTATAMN	20.55	4.66	4.07e-12
Barx1-Homer	AAACMATTAN	26.48	8.47	1.80e-11
FOXA1-Homer	WAWGTAAACA	31.99	12.29	2.98e-11
Hoxd11-Homer	NSYMATAAAA	30.08	11.23	6.99e-11
TEAD2-Homer	SCWGGAATGY	29.45	10.81	7.23e-11
Fox:Ebox-Homer	NNNVCWGWGYAAACAVN	36.44	15.89	8.97e-11
GSC-Homer	RGGATTAR	24.79	7.84	9.75e-11
Pitx1-Jaspar	YTAATCCH	24.36	7.63	1.19e-10
FOXO4-Jaspar	GTAAACA	33.05	13.56	1.61e-10

Table S1. Top 50 motifs significantly enriched in increased ATAC peaks relative to unchanged ATAC peaks at 30 minutes. Results generated using AME (McLeay and Bailey 2010).

bioRxiv preprint doi: https://doi.org/10.1101/2023.07.03.547524; this version posted July 3, 2023. The copyright holder for this preprint (which was not certified by peer review) is the author/funder, who has granted bioRxiv a license to display the preprint in perpetuity. It is made available under aCC-BY 4.0 International license.

Motif	Consensus	Percent decreased	Percent unchanged	Padj
Pax8-Homer	GYCAYGCHTGRCTGV	30.56	0.00	2.18e-3
Etv2-Homer	NNRYTTCCKGNN	41.67	5.56	1.05e-2
RORB-Jaspar	AWWTRGGTCAH	30.56	2.78	1.77e-2
Nr2f2-Secondary-Uniprobe	CKMKCSGGGTCAVBNH	22.22	0.00	2.00e-2
ERG-Homer	ACAGGAAGTR	36.11	5.56	2.46e-2
EKLF-Homer	NWGGGTGTGGCY	22.22	0.00	3.24e-2
Ascl1-Homer	NNVVCAGCTGBN	66.67	27.78	3.79e-2
Rara-Secondary-Uniprobe	MKMGYSGGGTCAMKBN	19.44	0.00	5.52e-2
RUNX1-Jaspar	BBYTGTGGTTT	50.00	16.67	6.26e-2

Table S2. Motifs significantly enriched in decreased ATAC peaks relative to unchanged ATAC peaks at 30 minutes. Results generated using AME (McLeay and Bailey 2010).
